# A novel toolbox to record CLE peptide signaling

**DOI:** 10.3389/fpls.2024.1468763

**Published:** 2024-08-14

**Authors:** Yong Zhou, Jie Zheng, Hao Wu, Youxin Yang, Huibin Han

**Affiliations:** ^1^ Guangdong Provincial Key Laboratory of Utilization and Conservation of Food and Medicinal Resources in Northern Region, Shaoguan University, Shaoguan, China; ^2^ College of Bioscience and Bioengineering, Jiangxi Agricultural University, Nanchang, Jiangxi, China; ^3^ Key Laboratory of Crop Physiology, Ecology and Genetic Breeding, Ministry of Education, Jiangxi Agricultural University, Nanchang, China; ^4^ Jiangxi Provincial Key Laboratory for Postharvest Storage and Preservation of Fruits & Vegetables, Jiangxi Agricultural University, Nanchang, China

**Keywords:** CLV3 peptide, fluorescent labeling, photoactivation, endocytosis, binding specificity

## 1 Introduction

In *Arabidopsis thaliana* genome, a multitude of small coding genes have been identified, with 200 of them are likely to encode small signaling peptides (Takahashi et al., 2019). Small signaling peptides, typically comprising fewer than 100 amino acids, mediate cell-to-cell communications, and are also pivotal for plant growth and responses to biotic and abiotic stressors (Oh et al., 2018; Olsson et al., 2019; Han et al., 2022; Xie et al., 2022; Bashyal et al., 2024; Datta et al., 2024; Wen et al., 2024). The *CLAVATA3*/*EMBRYO-SURROUNDING REGION* (*CLE*) genes encode proteins with a signal peptide at the N-terminus, a variable central domain, and highly conserved CLE motifs at the C-terminus (Fletcher, 2020; Khan et al., 2021). Following proteolytic processing and post-translational modifications (PMTs) (Stührwohldt and Schaller, 2019), the functional CLE motif, consisting of 12 to 14 amino acids, is secreted to the apoplast via the endoplasmic reticulum (ER) and Golgi apparatus, where it executes its biological functions. A plethora of evidence underscores the pivotal roles of CLE peptides as key modulators of cell proliferation and differentiation in the shoot apical meristem (SAM) (Schlegel et al., 2021; Song et al., 2021; Wang et al., 2022; Takahashi et al., 2023), root apical meristem (RAM) (Stahl et al., 2009; Gutiérrez-Alanís et al., 2017; Berckmans et al., 2020; Breiden et al., 2021), and vascular cambium (Yuan and Wang, 2021; Carbonnel et al., 2023; Diaz-Ardila et al., 2023). The CLE peptides also mediate cellular responses to various environmental clues (Xie et al., 2022; Bashyal et al., 2024; Datta et al., 2024).

CLE peptides function as local or long-distance signaling molecules, and can interact with the extracellular domains of plasma membrane (PM)-localized leucine-rich-repeat receptor-like kinases (LRR-RLKs), and the CLE-receptor pair thus regulates a wide range of plant developmental and adaptive processes (Narasimhan and Simon, 2022; Xie et al., 2022; Furumizu and Aalen, 2023; Bashyal et al., 2024; Datta et al., 2024). Extensive *in vitro* investigations employing isolated extracellular domains of LRR-RLKs have demonstrated the binding dynamics between CLE peptides and their corresponding (co)receptors, revealing differential binding affinities across distinct LRR-RLK subfamilies (Zhang et al., 2016a, 2016b; Guo et al., 2010; Crook et al., 2020). Alteration of the C-terminal anchorage site of CLE peptides can significantly diminish their receptor binding efficacy (Li et al., 2017). Moreover, exchanging one or more N-terminal residues between INFLORESCENCE DEFICIENT IN ABSCISSION (IDA) and CLE9 peptides has been shown to reduce their cognate receptor affinity (Roman et al., 2022). Therefore, both the C-terminal and N-terminal residues of CLE motif are required for receptor interactions, with the N-terminal residues conferring binding specificity (Zhang et al., 2016a, 2016b; Guo et al., 2010; Li et al., 2017; Crook et al., 2020; Roman et al., 2022). However, these *in vitro* assays do not fully reflect *in vivo* conditions or may contradict the *in vitro* binding results (Shinohara and Matsubayashi, 2015). Hence, elucidating the intracellular dynamics and binding specificity of CLE peptides *in vivo* remains a challenge. Recently, a new toolbox has been developed to facilitate *in vivo* analysis of CLV3-receptor interactions specificity and to capture the spatiotemporal dynamics of CLV3 peptides (Narasimhan et al., 2024). This innovative tool offers new perspectives for future functionality research on small signaling peptides.

## 2 Design of fluorescently conjugated CLV3 peptide

Over the decades, a diverse array of small-molecule fluorescent probes has been created (Zhu et al., 2016; Pramanik and Das, 2021; Yin et al., 2021), and are extensively utilized in plants (Iwatate et al., 2020; Yagi et al., 2021). These fluorescent probes are highly sensitive and specific, rendering them indispensable for visualizing subcellular structures such as the plasma membrane (PM), endoplasmic reticulum (ER), and vacuole (Zhu et al., 2016; Yagi et al., 2021). The fluorescent probes have also been engineered to elucidate the impacts of phytohormones on plant growth and development as well as their subcellular dynamics (Lace and Prandi, 2016; Balcerowicz et al., 2021). Various fluorescent labeled phytohormones, including auxin (Hayashi et al., 2014; Sokołowska et al., 2014; Pařízková et al., 2021), cytokinin (Nishikawa et al., 2000; Kubiasová et al., 2020), abscisic acid (Asami et al., 1997; Yamazaki et al., 2003), strigolactone analogs (Prandi et al., 2013; Yao et al., 2018; Van Overtveldt et al., 2019), jasmonic acid (Liu et al., 2012; Liu and Sang, 2013), brassinosteroid (Irani et al., 2012), and gibberellin (Shani et al., 2013), have been developed for this purpose. It is worth noting that, the 5(6)-carboxyfluorescein (FAM) fluorescence probe has been harnessed to tag CLE25/CLE26 and CLE45, facilitating the examination of their respective roles in drought response and vascular tissue development (Endo et al., 2019; Endo and Fukuda, 2024).

**Figure 1:**
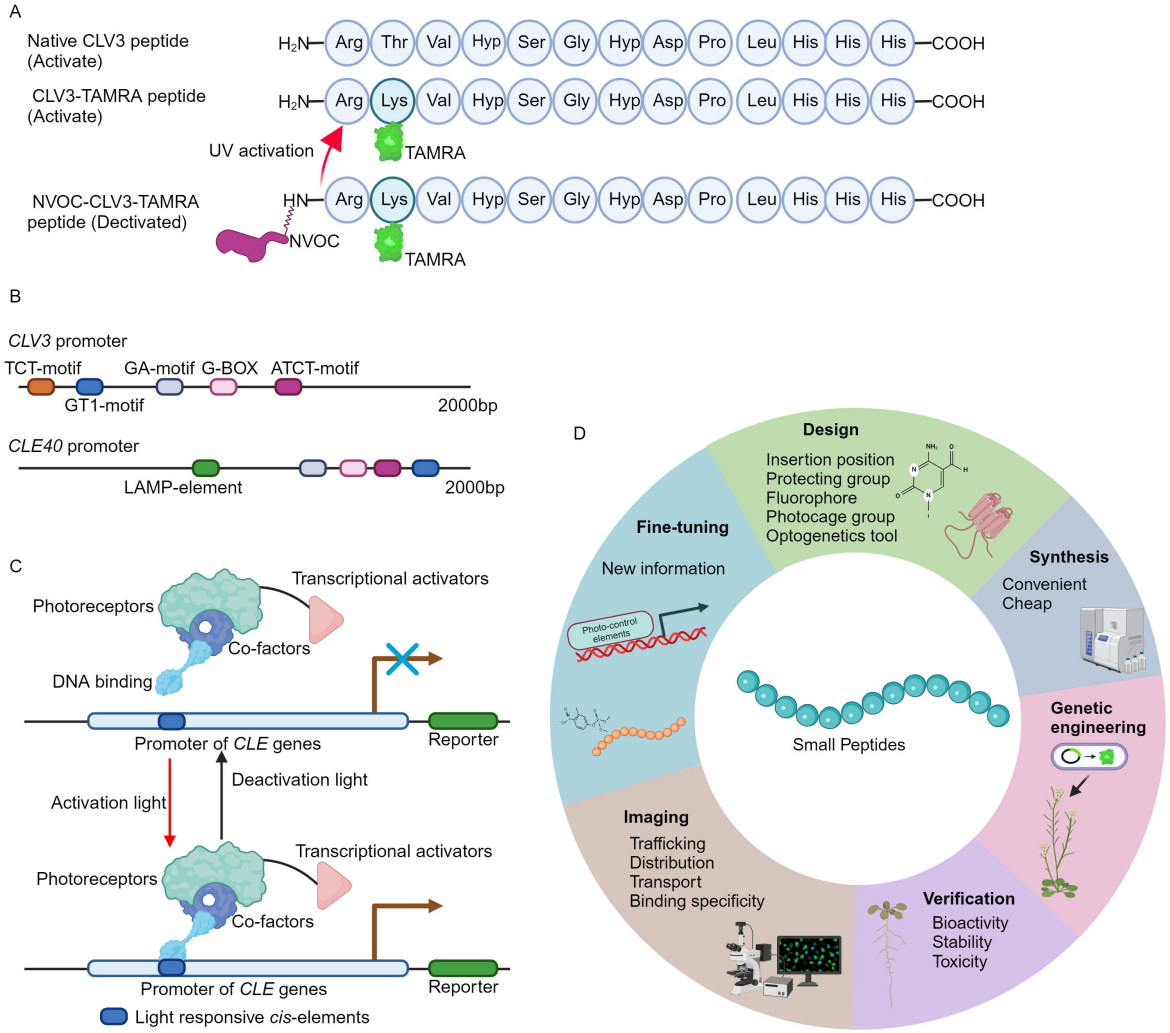
The development of fluorescence tagging and photo-activation of CLV3 peptide. **(A)** An overview of the structure of fluorescence labeling and photo-stimulation of the CLV3 peptide. **(B)** Numerous light-responsive *cis*-regulatory elements have been identified within the promoter regions of the *CLV3* and *CLE40* genes. The 2000 bp promoter sequences of *CLV3* and *CLE40* are obtained from TAIR 10 (https://www.arabidopsis.org/), and the PlantCARE database (https://bioinformatics.psb.ugent.be/webtools/plantcare/html/) is utilized to predict *cis*-regulatory elements. **(C)** An example of CLE pathway modulation through optogenetic techniques. Optogenetic engineering of CLE signaling involves the integration of photoreceptors, transcriptional activators, and DNA-binding domains into a functional complex. Upon exposure to specific activating wavelengths of light, this complex can interact with light-responsive elements within the promoter regions of *CLE* genes via its DNA-binding domains, thereby activating CLE signaling. Conversely, when exposed to deactivating wavelengths of light, the complex remains inactive, effectively terminating CLE signaling. **(D)** A summary of the basic principles for design of the fluorescence tagging and photo-activation of small peptides from other families.

Recently, a novel fluorescent labeled CLV3 peptide is successfully synthesized using a well-established solid phase peptide synthesis method (Wellings and Atherton, 1997; Narasimhan et al., 2024). In brief, the carboxyl terminus of the amino acid is *in situ* activated to form an active ester, facilitating its conjugation to a resin-bound amine group. Following the coupling, the fluorenylmethyloxycarbonyl (Fmoc) protecting group is selectively removed by piperidine, thus exposing the N-terminus for subsequent amino acid attachment. Through such iterative coupling cycles, the desired polypeptide sequence is assembled. Finally, the peptide is released from the resin, typically under acidic conditions, which also removes any protecting groups on the side chains (Wellings and Atherton, 1997; Narasimhan et al., 2024). Notably, the threonine at position 2 in CLV3 motif is replaced by lysine (**Figure 1A**) (Endo et al., 2019; Endo and Fukuda, 2024), as this amino acid is less conserved and can be modified without strongly influencing peptide activity (Ogawa et al., 2008; Song et al., 2012; Zhang et al., 2016a; Li et al., 2017). The lysine amino acid allows for the attachment of allyloxycarbonyl (Alloc) protecting group, which can be cleaved under specific conditions (Wojcik et al., 2012). Release of Alloc group leads to the introduction of the fluorophore 5-carboxytetramethylrhodamine (TAMRA) or fluorescein isothiocyanate (FITC) to the N-terminal of CLV3 peptide. Upon final cleavage, the fully deprotected CLV3-TAMRA and CLV3-FITC peptides are successfully synthesized (**Figure 1A**) (Narasimhan et al., 2024).

## 3 CLV3-TAMRA peptide targets canonical CLE signaling pathway

A root length assay is performed to evaluate the bioactivity of CLV3-TAMRA and CLV3-FITC peptides. The synthesized native CLV3 peptide significantly inhibits root growth (Fiers et al., 2005; Blümke et al., 2021), whereas the CLV3-TAMRA peptide necessitates a higher concentration to exert a similar suppressive effect on root growth (Narasimhan et al., 2024). Notably, the CLV3-FITC peptide exhibits no obvious impact on root growth. Furthermore, the *crn* mutant shows no response to both synthesized native CLV3 and CLV3-TAMRA peptides, indicating that the CLV3-TAMRA peptide operates via the canonical CLE signaling pathway, albeit with low efficacy (Fletcher, 2020; Khan et al., 2021; Narasimhan et al., 2024). The differential bioactivity between the CLV3-TAMRA and CLV3-FITC peptides is likely attributable to structural variations in their fluorophores. The hydroxyl groups present in FITC might interact with the peptide backbone, thereby affecting its conformation and impeding receptor binding (Narasimhan et al., 2024).

Upon ligand binding, the receptors usually undergo endocytosis followed by lysosomal degradation, a process crucial for modulating signal transduction and specificity (Claus et al., 2018). After perception of CLE peptides, the CLAVATA1 (CLV1) receptor is internalized in the SAM (Nimchuk et al., 2011; Somssich et al., 2015; Wang et al., 2023). CLV3-TAMRA signal is detected at the cell plasma membrane in Arabidopsis roots and SAM, the signal then accumulates increasingly in the vacuole through the clathrin-mediated endocytosis (CME) trafficking pathway. Additionally, CLV3-TAMRA peptide treatment induces the endocytosis of CLV1 and BAM1 receptors but not PEPR1 receptor, suggesting the binding specificity of CLV3-TAMRA (Guo et al., 2010; Roman et al., 2022; Wang et al., 2023; Narasimhan et al., 2024).

In conclusion, the fluorescence labeled CLV3-TAMRA retain bioactivity, can be used to inspect the subcellular dynamics of CLV3 peptides in the cells and its binding specificity *in vivo*.

## 4 Photoactivation of CLV3 peptide

Caged compounds are usually chemically or biologically active molecules that are deactivated by coupling to a photocleavable protecting group (caging group), and can be rapidly released upon UV illumination (Ellis-Davies, 2007; Li et al., 2023). The caged molecules have their unique properties. For instance, the capability for rapid intracellular uncaging, the spatial and temporal resolution can be precisely controlled by optical instrumentations, and intracellular concentration is tightly controlled through modulation of light intensity and duration (Ellis-Davies, 2007; Li et al., 2023). The nitroveratryloxycarbonyl (NVOC) group, belonging to the 4,5- dimethoxy-2-nitrobenzyl (DMNB) type, is one of the most frequently used cages (Kohl-Landgraf et al., 2014; Kneuttinger, 2022). This approach has successfully yielded DMPNB-caged auxin (Kusaka et al., 2009). In order to attain spatiotemporal regulation of CLV3 peptide activity, the NVOC group is conjugated to the N-terminus of the CLV3-TAMRA peptide, resulting in a fluorescently tagged and photoactivatable NVOC-CLV3-TAMRA peptide (**Figure 1A**) (Narasimhan et al., 2024).

In the absence of UV exposure, the NVOC-CLV3-TAMRA peptide exhibits no impact on root growth. Upon UV irradiation, the NVOC-CLV3-TAMRA peptide suppresses root growth in wild-type Arabidopsis seedlings, but not in the *clv2* mutant, indicating that the NVOC group is effectively cleaved, thereby releasing CLV3 peptide to repress root growth through the well-established CLE signaling pathway (Fletcher, 2020; Khan et al., 2021; Narasimhan et al., 2024). Additionally, fluorescence signals are predominantly localized in the apoplastic space before photoactivation in NVOC-CLV3-TAMRA peptide-treated Arabidopsis roots. After UV photoactivation, the fluorescence signals are observed at the plasma membrane, endosomal compartments, and vacuoles, suggesting a photo-activated trafficking of CLV3 peptide in the target cells (Narasimhan et al., 2024).

## 5 Future perspectives

The pioneering approach of fluorescence tagging and photo-activation of the CLV3 peptide, alongside previously developed FAM and CdTe quantum dots (QDs) labeled CLE peptides (Yu et al., 2014; Endo et al., 2019; Endo and Fukuda, 2024; Narasimhan et al., 2024), provides crucial insights into the functional elucidation of CLE peptides. The CLV3-TAMRA and NVOC-CLV3-TAMRA peptides are easy to synthesis, stable, non-toxic to plants, and can be utilized to investigate the subcellular dynamic of CLV3 peptide and its receptor binding specificity *in vivo* (Yu et al., 2014; Narasimhan et al., 2024). Many different fluorescence groups have been created (Zhu et al., 2016; Pramanik and Das, 2021; Yin et al., 2021), however, not all of these fluorophores preserve their bioactivity when conjugated to the CLV3 motif (Narasimhan et al., 2024). Therefore, it is advisable to conduct computational modeling to mitigate their effects on peptide conformational alterations and receptor binding interactions. On the other hand, genetically engineered photo-activated CLE signaling systems remain undeveloped. In the promoter region of *CLE* genes, exampled by *CLV3* and *CLE40*, multiple light responsive *cis*-regulatory elements can be identified (**Figure 1B**), suggesting the potential for genetic (de)activation via optogenetics. Over recent decades, a multitude of light receptors or sensors from bacteria, fungi, and plants have been discovered and integrated into optogenetic tools (Kolar et al., 2018; Zhang et al., 2024). These optogenetic systems have been successfully introduced into living cells to control spatio-temporal gene expression, protein stability, subcellular localization, and receptor activity through optical means (Banerjee and Mitra, 2020; Christie and Zurbriggen, 2021; Shikata and Denninger, 2022; Konrad et al., 2023). Additionally, these diverse optogenetic systems have been applied to visualize the subcellular distribution and dynamics of plant phytohormones such as auxin (Jásik et al., 2013; Salanenka et al., 2018) and gibberellin (Rizza et al., 2017). Based on the well-established design principles and optogenetic frameworks (Kolar et al., 2018; Banerjee and Mitra, 2020; Zhang et al., 2024), the engineering of genetic activation incorporating the promoters of *CLE* genes by a specific light wavelength will be achievable (**Figure 1C**), thereby spatio-temporally control CLE peptide signaling. Particularly, the plant usable light-switch elements (PULSE) system, which enables plant growth under normal light conditions, and is exclusively activated by red light stimuli (Ochoa-Fernandez et al., 2020).

Plants can produce various small signaling peptide families (Olsson et al., 2019; Takahashi et al., 2019). Among these small peptides, for example, C-TERMINALLY ENCODED PEPTIDE (CEP) is capable of being translocated from roots to aerial parts through long-distance transport mechanisms (Tabata et al., 2014). Remarkably, this long-distance translocation is achieved through the engineering of the Alexa Fluor 488 conjugated CEP1 peptide (Ohkubo et al., 2017). Subsequently, the leaf localized CEP RECEPTORs (CEPRs) can recognize CEP peptides, thus modulating plant development and stress responses accordingly (Taleski et al., 2024). Notably, the FITC conjugated Medicago CEP1 peptide is utilized to examine the ligand-receptor specificity between CEP receptors (Lee et al., 2021). Nevertheless, elucidating the intracellular localization and cellular dynamics of the majority of small peptides triggered by specific developmental cues or environmental stimuli, as well as their receptor binding affinities *in vivo*, still remains a significant challenge. In accordance with the methodologies for the synthesis of fluorescently labeled and photoreactive CLE peptides as well as CEP peptides (**Figure 1D**) (Yu et al., 2014; Ohkubo et al., 2017; Endo et al., 2019; Lee et al., 2021; Endo and Fukuda, 2024; Narasimhan et al., 2024), it is plausible to design and synthesize a variety of fluorescence-labeled and photo-activatable bioactive peptides derived from other small peptide family. These innovative tools will enable a more detailed investigation into the elusive biological functions of small signaling peptides in agronomic and horticultural plants, thus advancing the application of small peptides in modern agriculture.
